# Hb Santa Ana异常血红蛋白病1例

**DOI:** 10.3760/cma.j.issn.0253-2727.2023.06.010

**Published:** 2023-06

**Authors:** 小凤 魏, 璇 商

**Affiliations:** 1 南方医科大学基础医学院，医学遗传学教研室，广州 510515 Department of Medical Genetics, Southern Medical University, College of Basic Medical Sciences, Guangzhou 510515, China; 2 南方医科大学南方医院，地中海贫血诊治创新研究中心，广州 510515 Innovation Center for Diagnostics and Treatment of Thalassemia, Nanfang Hospital, Southern Medical University, Guangzhou 510515, China

患者，男，8岁，吉林长春人。患儿出生后新生儿期黄疸消退延迟，11月龄出现无明显诱因发热伴尿色加深，血常规：HGB 67 g/L，平均红细胞体积（MCV）92.7 fl，平均血红蛋白含量（MCH）29.0 pg，平均血红蛋白浓度（MCHC）313 g/L，网织红细胞比值13.91％，网织红细胞计数0.037×10^12^/L；肝功能：总胆红素（TBIL）19.8 µmol/L，直接胆红素（DBIL）12 µmol/L，间接胆红素（IBIL）7.8 µmol/L。外周血涂片：杆状核粒细胞2％，分叶核粒细胞26％，淋巴细胞62％，单核细胞6％，嗜酸分叶核粒细胞2％，异型淋巴细胞2％，已成熟红细胞大小不一，球形红细胞13％，易见泪滴状、盔甲状、棒状等异形红细胞碎片。血红蛋白电泳：HbA 69.9％，HbA_2_ 2％，HbF 18.4％，HbE 6.6％，Hb-S/D 3.1％，中国人常见17种β珠蛋白基因点突变检测提示β^N^/β^N^。自身免疫性抗体：IgM 14.9 U/ml，抗核抗体弱阳性，其余抗体阴性。骨髓涂片：增生活跃，红系增生旺盛，有核红细胞形态大致正常，偶见双核中幼红细胞，分裂红细胞易见，部分成熟红细胞中心淡染区缩小，部分成熟红细胞中空明显，可见变形，可见H-J小体，点彩红细胞易见。输注洗涤红细胞维持HGB为90～100 g/L，患儿尿色加深症状无改善。患儿4岁时出现肝脾肿大症状，腹部B超：肝右锁骨中线肋缘下2.4 cm，脾左肋缘下2.9 cm；尿常规：尿胆红素（++）。为明确诊断，患儿前来本实验室。签署知情同意书后抽取患儿及其父母外周血进行表型及基因型分析。检测结果提示患儿表现为大细胞性贫血，血常规：HGB 76 g/L，MCV 105.57 fl，MCH 30.08 pg，MCHC 276 g/L；高效液相色谱法行血红蛋白电泳：HbF 18.4％，HbA_2_ 2.0％，HbS 9.7％，利用本实验室建立的毛细管区带快速电泳法测定α和β珠蛋白链提示患儿有异常血红蛋白。患儿常规的地中海贫血基因检测阴性（3种中国人群常见的缺失型α地贫基因、3种中国人群常见的非缺失型α地贫基因和17种中国人群常见的β地贫基因），为了检测患儿是否存在未知突变，对整个β珠蛋白基因进行了DNA测序，测序结果提示患儿携带β珠蛋白基因的罕见错义突变CD88（T>C）。而患儿父母表现出正常的血液学表型：患儿父亲HGB 161 g/L、HbF 1.1％、HbA_2_ 3.0％，患儿母亲HGB 136 g/L、HbF 1.0％、HbA_2_ 2.5％；常规地中海贫血基因检测也均为阴性，且测序发现父母双方都没有携带患儿所携带的罕见的错义突变CD88（T>C）。考虑患儿是由于自发突变引起的HBB基因突变，经查询数据库该突变引起异常血红蛋白Hb Santa Ana，该异常血红蛋白案例在国外有过报道，该突变携带患者多表现出大细胞性贫血伴随异常血红蛋白。对患儿进行随访，目前血红蛋白维持在90～100 g/L，肝脾肿大未有改善。

